# Gallic Acid Alleviates Injury of Intestine Induced by Escherichia Coli: Protective Role of Metalloproteinase and Antioxidants on Small Intestine In-vivo

**DOI:** 10.31661/gmj.v13i.3375

**Published:** 2024-08-10

**Authors:** Muhammad Halwani

**Affiliations:** ^1^ Department of Microbiology, Al Baha University, Al Baha, Saudi Arabia

**Keywords:** Escherichia Coli, Gallic Acid, Anti-inflammatory, Antioxidant, Metalloproteinase

## Abstract

Background: Escherichia coli (E. coli) is a common pathogen that can cause significant morbidity and mortality in hospitalized patients. The aim of this study was to investigate the effects of gallic acid (GA) on a mice infected with of E. coli enteritis and evaluate the serum levels of interleukin-6 (IL-6) and matrix metalloproteinase (MMP)-9, as well as any histopathological changes before and after exposure. Materials and Methods: Forty Swiss male mice were divided into four groups: Group I (negative control), Group II (received oral GA, 80 mg/kg/b.wt), Group III (orally inoculated with E. coli, 1×107 CFU, for four days), and Group IV (received oral GA, 80 mg/kg/b.wt, for 10 days after E. coli inoculation). Serum was collected to assess IL-6 and MMP-9 levels. Intestinal samples were examined for antioxidant parameters, including superoxide dismutase (SOD), glutathione peroxidase (GSH-Px), and catalase. Histopathology and immunohistochemistry were performed. Results: Group III exhibited significantly higher IL-6 and MMP-9 levels compared to the other groups (P0.001). Antioxidant activity in the intestine, measured by SOD and GSH-Px, was lower in Group III compared to Group I. Conversely, Group IV showed significant improvements in biochemical, histopathological, and immunohistochemical outcomes, alongside reduced intestinal damage caused by E. coli. Conclusion: This study demonstrates that E. coli infection in mice increases IL-6 and MMP-9 levels while decreasing intestinal antioxidants. Concurrent administration of GA significantly improves outcomes, suggesting its potential as a therapeutic remedy for E. coli-induced intestinal damage. Furthers research is imperative to determine the underlying pathways by which GA exerts its beneficial outcomes.

## Introduction

Escherichia coli (E. coli) is a pathogenic organism responsible for causing infectious diarrhea in adults and children. It is one of the primary causes of death among children in developing countries [1]. The global epidemiological significance of E. coli infections is considerable and warrants ongoing attention as a prominent public health problem [2]. E. coli possesses the ability to produce and secrete various virulence factors, including adhesins and enterotoxins [3]. The pathogenesis of E. coli infection involves the utilization of colonization factors, known as adhesins, to facilitate bacterial adherence to intestinal epithelial cells. Once attached to the intestine, the bacteria initiate their pathogenic processes by secreting toxins [4]. This disruption of intestinal barriers contributes to the development of diarrhea, showcasing the complex mechanisms by which E. coli adhesins and pathogenic factors contribute to the infection process [4][5].

Certain natural compounds have exhibited promising effects on E. coli, providing potential alternatives that may offer greater advantages [6]. Examples of such compounds include allicin in garlic [7], honey [8], and curcumin [9]. These natural compounds have demonstrated antimicrobial and anti-inflammatory properties, which can aid in combating E. coli pathogenicity in the intestine.

Gallic acid (GA), a natural polyphenol compound found in various edible plants and botanicals, has been extensively studied for its biological and pharmacological activities [9]. GA has been shown to possess anti-inflammatory, antimicrobial, anticancer, antioxidant, gastroprotective, neuroprotective, and cardioprotective effects [10]. Researchers have found that GA interferes with intra-cellular inflammatory pathways that contribute to the development of ulcerative colitis [11]. Researchers have also found that GA can interfere with intra-cellular inflammatory pathways because of its contribution to the development of ulcerative colitis. GA mechanism of action can inhibit the expression of nuclear transcription factors such as signal transducer and activator of transcription 3 (STAT3), and nuclear factor-κB (NF-κB), which have been implicated in the induction of inflammatory responses [12].

Additionally, GA suppresses both the production and activity of pro-inflammatory mediators (e.g., TNF-α, INF-γ, IL-1β, IL-6, IL-17, IL-21, IL-23, COX-2, i-NOS) and decreases the infiltration of neutrophils and CD68+ macrophages in the colon [12]. Furthermore, it can inhibit the expression of nuclear transcription factors, including NF-κB and STAT3, which are implicated in inflammatory processes [12]. Additionally, GA also decreases the synthesis and activity of pro-inflammatory cytokines and proteins (e.g., TNF-α, INF-γ, IL-1β, IL-6, IL-17, IL-21, IL-23, COX-2, i-NOS). Finally, it can also decrease the infiltration of CD68+ macrophages and neutrophils in the colon [13].

This study’s primary aim is to investigate GA’s therapeutic potential in mice infected with E. coli-induced enteritis, with a focus on its antioxidant and anti-inflammatory consequences. Specifically, this research will investigate GA’s efficacy in reducing inflammation, tissue damage, and oxidative stress, in the intestine, as measured by histopathology and immunohistochemistry analysis. The study will also monitor serum levels of MMP-9 and IL-6 to assess GA’s potential as a biomarker for inflammatory conditions. Ultimately, it aims to elucidate the mechanisms by which GA exerts its protective effects against E. coli-induced enteritis.

## Material and Methods

Animals

This study adhered to all relevant ethical guidelines and regulations. Moreover, its experimental procedures are documented in accordance with the Association for Research in Veterinary Science and Education (ARRIVE) guidelines [14]. Ethical approval was obtained from the Ethical Committee and Scientific Research, Faculty of Medicine, University of Al-Baha (REC/MIC/BU-FM/2023/99). Forty adult male Swiss Albino mice, weighing 22 ± 2 grams at the start of the experiment, and aged between 7 and 9 weeks old were used in this study. The samples were sourced from the Animal House at King Abdulaziz University in Jeddah, Saudi Arabia. They were maintained in a controlled environment: appropriate temperature, a moderate humidity level of 60% ± 5% with a 12-hour light/dark cycle. These mice had an unlimited supply of food and water in the form of pellets from Oriental Chow during the trials. To eliminate any potential biases that might arise from sex-related differences, male mice were exclusively used and then categorized into four distinct groups. The use of Swiss Albino mice in this study is justified due to their common use in laboratory research, which is attributed to their docile nature, ease of handling, and genetic uniformity.

Isolation, Identification and the Lethal Dose of the Bacteria

The bacterial isolate was received by the microbiology laboratory at the Faculty of Medicine, University of Al-Baha from Prince Meshari Hospital, Baljurashi City, Al-Baha, Saudi Arabia. The isolate was obtained from a clinical sample of a hospitalized individual with diarrhea as the primary symptom. The sample was cultured on Salmonella Shigella agar and MacConkey agar according to hospital standards. After an incubation period of 24 hours, the MacConkey agar plate displayed growth of a lactose-fermenting organism. Comprehensive full identification and sensitivity testing were carried out using the VITEK 2 system (bioMérieux, France), which conclusively identified the organism as E. coli and revealed it to be a non-multidrug-resistant strain [15][16].

E. coli Lethal Dose 50 (LD50)

The lethal dose (LD50) of a substance is a measure of the amount that causes 50% mortality in test animals when administered simultaneously. This parameter is used to evaluate the acute toxicity of a material, which has the potential for short-term poisoning. In this study, the LD50 of E. coli was determined and given by oral inoculation to the mice three times a week at various doses of viable E. coli suspended in phosphate-buffered saline (PBS).

A total of eight doses were tested: 1×10³, 1×104, 1×105, 1×106, 1×107, 1×108, 1×109, and 1×1010 CFU. Six mice were used for each dose group, and six additional mice served as a control group receiving PBS alone. The results showed that the dose 1×109 was responsible for inducing 50% mortality in the mice group, whereas the dose 1×107 did not result in any mortality. The preliminary studies conducted prior to the experiment found that this dose effectively infected mice without causing death [17].

Gallic acid and Lethality Study in Mice

Gallic acid (3,4,5-trihydroxybenzoic acid) was obtained from Sigma-Aldrich, Inc., St. Louis, MO, USA. The GA was dissolved in water and prepared in distilled water (DW) at various concentrations according to the intended use. A total of four groups of six mice each were formed and administered oral sterilized GA at concentrations of 50, 100, 150, 200, and 300 mg/kg body weight. The number of live mice was counted daily for a period of 30 days, in compliance with ethical guidelines. Following the oral administration of GA to six mice, no discernible effects were observed in any of the tests, and the GA was deemed safe for administration.

Experimental Design

A total of four groups of ten mice each were created using random selection from the sample population. To ensure equal sample sizes, the block randomization method was employed, which involved randomly assigning participants to groups with equal sample sizes (same source, sex, date of birth, and median weight). This method ensures that sample sizes for all groups are balanced over time [18]. The reference group was designated as Group I. Group II received a predetermined dose of 80 mg/kg body weight (b.wt) of GA orally until the end of the experiment, which was completed after 21 days. Group III was given an oral inoculation of 1×107 CFU of E. coli in 0.3 mL of phosphate-buffered saline (PBS) without any treatment. Group IV received an oral inoculation of 1×107 CFU of E. coli on day 4. It was treated orally; 80 mg/kg b.wt of GA in 0.3 mL distilled water for ten days, starting on day 4. Following the final treatment, all experimental groups were humanely euthanized using ether anesthesia, and subsequently subjected to cardiac puncture for the collection of blood samples. Blood was collected and subsequently allowed to coagulate, following which it was centrifuged at 3000 revolutions per minute for a duration of 10 minutes. The serum was separated and stored at -20°C until analysis for interleukin-6 (IL-6) and metalloproteinase-9 (MMP-9). The intestine was removed, cleaned, and prepared for the measurement of antioxidant parameters, including superoxide dismutase (SOD), glutathione peroxidase (GSH-Px), and catalase (CAT). The remaining intestine was preserved in 10% formalin for histopathology and immunohistochemistry analysis. By using the protocol outlined by El Naaa et al., the concentration of interleukin-6 (IL-6) in mouse serum was established [19]. This method involves mixing 100 μl of anti-IL-6 immunoglobulin G labeled with horseradish peroxidase with 20 μl of diluted serum samples in 10 mmol/l ethylenediaminetetraacetic acid. The mixture is then incubated at 37°C for 60 minutes, followed by washing and the addition of a reagent to measure the microplate-bound horseradish peroxidase activity. The absorbance is measured at 450 nm, and the amount of IL-6 in the serum is determined through a standard curve.

The level of matrix metalloproteinase (MMP-9) in mouse serum was quantified using a competitive enzyme-linked immunosorbent assay (ELISA) from Cytoimmune Science Inc., MD [20]. Blood samples were taken from all animal groups and allowed to clot before centrifugation and extraction of serum (preserved at -70°C). The ELISA kit was used to determine MMP-9 levels, which involves pre-coating an MMP-9-specific monoclonal antibody onto a microplate, followed by addition of standards and samples, and subsequent binding with an enzyme-linked polyclonal antibody. The reaction is stopped, and the color intensity is measured at 450 nm.

Measurement of Intestine Homogenate’s SOD, GSH-Px and CAT Activities

In all mice groups, the activities of glutathione peroxidase (GSH-Px), superoxide dismutase (SOD), and catalase (CAT) in intestinal homogenates were measured. The intestines were homogenized in phosphate-buffered saline. They were then centrifuged to obtain the supernatant, which was used to measure the enzyme activities. Through Nishikimi et al.’s method, SOD activity was measured [21]. This involved adding the sample to a cuvette with sodium pyrophosphate buffer, nitro blue tetrazolium, and NADH, and measuring the absorbance increase at 560 nm. Using the Prins and Loose method, the GSH-Px activity was measured [22], which involves adding the homogenate to a tube containing tungstate solution and then adding 5.5-dithio-bis-(2-nitrobenzoic acid reagent and measuring the optical density at 412 nm. The cuvette was then treated with Hydrogen Peroxide (H2O2) (7722-84-1 - Calbiochem, Sigma-Aldrich, Inc., St Louis, MO, USA) in increments until the final concentration reached 10 mM. The absorbance at 240 nm was used to measure the disappearance of H2O2. CAT activity was measured using Clairborne’s methodology [23]. The process involves combining the supernatant with a mixture of phosphate buffer and H2O2in a cuvette, and then monitoring the reduction of the H2O2 levels at 240 nm.

Immunohistochemistry and Histopathology

To prepare paraffinized sections for immunohistochemistry, they were de-paraffinized through a series of solvents, including distilled water, xylene, and ethanol. The sections were then microwave-heated in citrate buffer to enhance antigen retrieval. Immunohistochemistry was performed using an Abcam Horseradish Peroxidase/ diaminobenzidine (HRP/DAB) Detection kit, with primary antibodies against IL-6 (ab9324) and TNF-α (ab220210). The antibodies were diluted at 1:400 and 1:100 respectively. Initially, the sections were treated with primary antibodies, which was then followed by the addition of (DAB) substrate and HRP conjugate. Finally, the sections were examined under a light microscope [24].

The intestinal organs of all mice groups were processed for histological examination. The organs were first cleaned with phosphate buffered saline (PBS), then fixed in 10% formalin and subsequently embedded in paraffin using the EG 1150H paraffin dispensing module. The slides were then deparaffinized and rehydrated to distilled water. The tissue was stained with Mayer’s Hematoxylin for one minute, followed by washing with tap water until the blue color ceased to fade. The blue nuclei were then stained with 1X PBS and washed with distilled water. The tissue was counterstained without rinsing and then dehydrated with ethanol and xylene. Finally, the slides were mounted, and cover slipped with a Hematoxylin and Eosin (H&E) stain. The sections were processed and stained according to a standard protocol used in histopathological laboratories [25][26].

Samples and Digital Image Analysis

Sample size calculation was based on pervious equivalent studies following Faul et al. [27]. Using the G*power software version 3.1.9.5 (Microsoft Corporation, USA), a sample size calculation was performed to determine the required size for each group based on the effect size of 1.6254, a two-tailed test, α error=0.05, and power=90.0%. The calculation yielded a sample size of 10 subjects per group. Slides were photographed with the MEIJI MX5200L light microscope’s MVV5000CL digital eyepiece installed, a 0.5 photo adaptor, and Future WinJoe software (Informer Technologies, Inc. Los Angeles, California. USA), using a 10X and 40X objective. The acquired 10X images were analyzed using Fiji ImageJ (version 1.51r; NIH, Maryland, USA) software in conjunction with the color deconvolution 2 plugin (histological dyes digital separation) on an Intel® core I7®-based computer, to calculate the percentage of immunohistochemical staining surface area.

The outcome was the acquisition of three distinct digital images: one stained with H&E, one immunohistochemically stained with DAB, and a complementary image. A total of five random areas (200x200 micrometers each) were analyzed on each slide. [28]. GraphPad Prism 8 (GraphPad Software, Insightful Science, San Diego, California, United States) was used to analyze the data fed into Microsoft Excel software(Microsoft Corporation, USA). Tests of normality (Shapiro-Wilk tests) demonstrated that immunohistochemical positive area percentages and biochemical measurements for numerical data were distributed normally. Data was presented as mean and standard deviation (SD) values [29].

Statistical Analysis

A one-way analysis of variance (ANOVA) followed by post hoc Tukey’s multiple comparison test was employed to investigate differences between normally distributed data sets. The strength of the linear relationships between variables was measured using Spearman’s rank correlation coefficient, which ranged from -1 (perfect negative correlation) to 1 (perfect positive correlation), allowing for the quantification of the degree of association between the variables. The statistical significance of the results was evaluated at 0.05 level [30].

## Results

**Figure-1:**
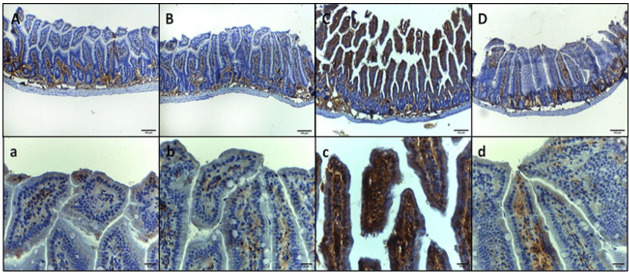
Figure 1. IL-6 immunohistochemical staining micrographs of intestinal sections of (A, a) Control negative group, showing mild immunoreactivity in the basal layer of mucosa with minimal reactivity at higher layers. (B. b) Control negative treated GA group, showing mild immunoreactivity in the basal layer of mucosa with minimal reactivity at higher layers. (C, c) Infected E. coli group, showing immunoreactivity throughout the entire mucosa (i.e., epithelium and lamina propria). (D, d) Infected E. coli treated GA group, showing mild immunoreactivity in the basal layer of mucosa with minimal to mild reactivity at higher layers: upper raw original magnification 100X, lower raw 400X and scale bar 100 μm and 20 μm respectively.

**Figure-2:**
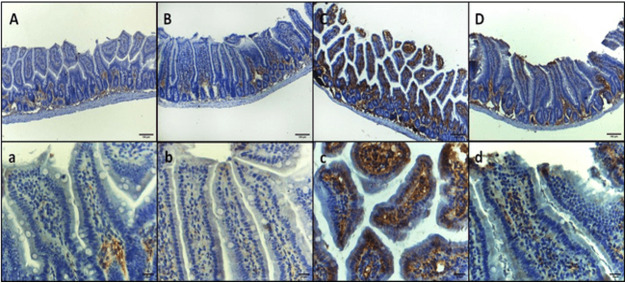
Figure 2. TNF-α immunohistochemical staining micrographs of intestinal sections of (A, a) Control negative group, showing mild immunoreactivity in the basal layer of mucosa with minimal reactivity at higher layers. (B. b) Control negative treated GA group, showing mild immunoreactivity in the basal layer of mucosa with minimal reactivity at higher layers. (C, c) Infected E. coli group, showing intense immunoreactivity in the epithelium and lamina propria in the whole mucosa. (D, d) Infected E. coli treated GA group, showing mild immunoreactivity in whole mucosa: upper raw original magnification 100X, lower raw 400X and scale bar 100 μm and 20 μm respectively.

**Figure-3:**
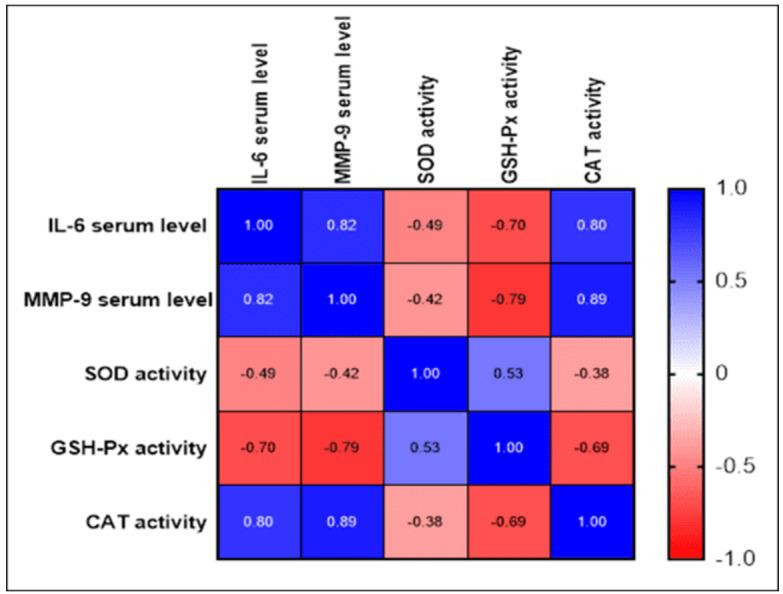
Figure 3. The findings of this study have shown there is a significant strong positive correlation between the IL-6 serum level, MMP-9 serum level and CAT activity, r=0.825, 0.798 (P-value<0.0001), a significant weak negative correlation between the IL-6 serum level and SOD activity, r=-0.489 (P-value=0.0014), and a significant moderate negative correlation between the IL-6 serum level and GSH-Px activity, r=-0.698 (P-value<0.0001). A weak negative correlation between the MMP-9 serum level and SOD activity, r=-0.419 (P-value=0.0071), a significant strong negative correlation between the MMP-9 serum level and GSH-Px activity, r=-0.791 (P-value=0.0004), and a significant strong positive correlation between the MMP-9 serum level and CAT activity, r=0.893 (P-value <0.0001).There is a significant moderate positive correlation between the SOD activity and GSH-Px activity, r=0.529 (P-value<0.0001), and a significant weak negative correlation between the SOD activity and CAT activity, r=-0.377 (P-value=0.0164). There is also a significant moderate negative relationship between the activities: GSH-Px and CAT, r=-0.686 (P-value<0.0001).

**Figure-4:**
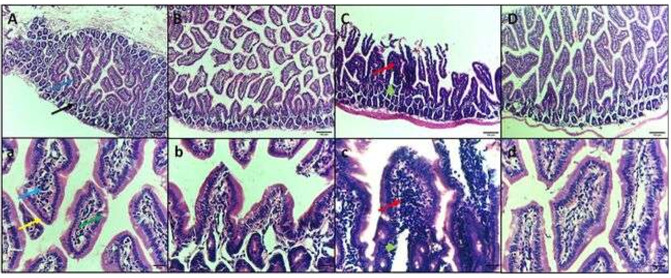
Figure 4. H∓E staining micrographs of intestine sections of (A, a) control negative group, showing normal intestinal structure with villi and crypts lined by columnar epithelium with brush border and goblet cells. (B. b) Control negative treated GA group, showing normal intestinal structure. (C, c) Infected E. coli group, showing abscess in the villus, and intense inflammatory cells infiltration. (D, d) Infected E. coli treated GA group, showing resolve of the inflammation and restoration of normal intestinal architecture. Black arrow: crypt, Dark blue arrow: villus, Light blue arrow: lamina propria, yellow arrow: columnar epithelium with brush border, Green arrow: goblet cells, Red arrow: inflammatory cells infiltration, Green arrow head: abscess. Upper raw original magnification 100X, lower raw 400X and scale bar 100 μm and 20 μm respectively.

**Table:** Table1. Serum IL-6 Level (pg/ml) and Serum MMP-9 level (ng/ml) in Different Mice Groups

**Group**	**Control Negative Group I **	**Control Negative Treated GA Group II **	**Infected *E. Coli* Group III **	**Infected *E. Coli* Treated GA Group IV **	**P Value**
**Serum Il-6 Level (pg/ml) Mean **	**24.32**	**24.68**	**48.49 ^ab^**	**26.04 ^abc^**	**P<0.0001** **F=1513**
**SD**	**±0.62**	**±1.23**	**±0.7**	**±1.13**	
**Serum MMP-9 Level (ng/ml) Mean **	**44.4**	**45.98**	**107.01 ^ab^**	**51.83 ^abc^**	**P<0.0001** **F=4533**
**SD**	**±1.12**	**±1.02**	**±1.69**	**±1.67**	

Each value is expressed as a mean with its ± SD **Test Used:** ANOVA followed by a post hoc Tukey’s multiple comparison test. **a:** significance vs. control group; **b:** significance vs. drug control group; **c:** significant vs. model group at P<0.05P<0.05

**Table:** Table2. SOD, GSH-Px and CAT Activity Level (U/mg protein) in Different Mice Groups.

**Group**	**Control Negative Group I **	**Control Negative Treated GA Group II **	**Infected *E. Coli * Group III **	**Infected *E. Coli * Treated GA Group IV **	**P Value**
SOD Activity (U/Mg Protein) Mean	40.92	41.86	23.29 ^ab^	42.14 ^ac^	P<0.0001 F=971.8
SD	±0.99	±0.91	±0.7	±1.08	
GSH-PX Activity (U/Mg Protein) Mean	95.48	95.94	41.89 ^ab^	83.88 ^abc^	P<0.0001 F=7736
SD	±0.72	±1.46	±0.64	±0.57	
CAT Activity (U/mg protein) Mean	4.77	5.04	14.44 ^ab^	5.35 ^c^	P<0.0001 F=701.2
SD	±0.19	±0.48	±0.95	±0.3	

Each value is expressed as a mean with its ± SD **Test Used:** ANOVA followed by a post hoc Tukey’s multiple comparison test. **a:** significance vs. control group; **b:** significance vs. drug control group; **c:** significant vs. model group at P<0.05P<0.05

Serum Analysis of IL-6 (pg/ml) and Metalloproteinase-9 (ng/ml) in Mice Groups Using Competitive Enzyme-linked Immunosorbent Assay (ELISA)

A competitive enzyme-linked immunosorbent assay (ELISA) was used to analyze the levels of interleukin-6 (IL-6) and metalloproteinase-9 (MMP-9) in the mice serum. IL-6 is a key mediator of inflammation, while MMP-9 is a protein that breaks down connective tissue.

The obtained results showed that mice treated with 80 mg/kg of GA had significantly reduced levels of IL-6 compared to infected mice. In the treated group, the IL-6 was 26.04 ± 1.13 pg/ml, which is close to the normal level. In contrast, the infected mice had an IL-6 level of 48.49 ± 0.70 pg/ml group III. The distinction between the two groups was found to be statistically significant (P<0.0001).

Similarly, the level of MMP-9 was significantly reduced in the treated group, with a level of 51.83 ± 1.67 ng/ml compared to the infected group (group III), which had a level of 107.01 ± 1.69 ng/ml. The variance between these two groups was statistically considerable (P<0.0001, Table-1).

Intestinal Tissue SOD, GSH-Px, and CAT Activity (unit/mg protein) in Mice Groups

Assessment of antioxidant enzyme activities in the intestinal tissue of mice revealed significant alterations in response to E. coli infection and treatment with galantamine (GA). In group III, mice inoculated with 1 × 107 CFU E. coli showed significantly decreased mean activities of superoxide dismutase (SOD), glutathione peroxidase (GSH-Px), and catalase (CAT) enzymes compared to normal mice (group I). In contrast, treated group IV, receiving 80 mg/kg/b.wt of GA, exhibited a significant boost in SOD and GSH-Px activity, as well as a reduction in CAT activity. Specifically, the mean activity of SOD, GSH-Px, and CAT enzymes in group IV was 42.14±1.08, 83.88±0.57, and 14.44±0.95 units/mg protein, respectively, which was significantly different from group III (23.29±0.70, 41.89±1.46, and 5.04±0.48 units/mg protein, respectively). Notably, the observed changes in SOD, GSH-Px, and CAT activity in group IV were sufficient to bring these values into insignificance compared to those in control group I (Table-2).

Immunohistochemical Examination

The immunohistochemical examination of intestinal sections revealed distinct patterns of interleukin-6 (IL-6) expression in each group. In group I, the normal mice, IL-6 immunoreactivity was observed in the basal layer of mucosa with minimal reactivity in the higher layers. Similarly, in group II, the GA-treated mice, mild immunoreactivity was detected in the basal layer of mucosa with minimal reactivity at higher layers. In contrast, group III, infected mice inoculated with 1×107 CFU E. coli, exhibited intense immunoreactivity throughout the entire mucosa, specifically in the epithelium and lamina propria. Group IV, infected mice treated with GA, demonstrated a return to the pattern observed in groups I and II, characterized by mild immunoreactivity in the basal layer of mucosa with minimal to mild reactivity at higher layers (Figure-1 A, B, C, and D, respectively). These findings suggest that E. coli infection induces a marked increase in IL-6 expression in the intestinal mucosa, which is attenuated by treatment with GA. The immunohistochemical staining of intestinal sections for tumor necrosis factor-alpha (TNF-α) revealed distinct patterns of expression in each group. In group I, the control negative mice, and group II, the GA-treated mice, mild immunoreactivity was observed in the basal layer of mucosa with minimal reactivity at the higher layers. In contrast, group III, infected mice inoculated with 1×107 CFU E. coli, exhibited intense immunoreactivity throughout the entire mucosa (i.e., epithelium and lamina propria). Group IV, infected mice treated with GA, showed a decreased expression of TNF-α, characterized by mild immunoreactivity in the whole mucosa (Figure-2, A, B, C, and D). To further analyze the results, the integrated density of IL-6 and TNF-α intestine staining for each group was assessed (Table-3). Significant correlations were evident between the levels of matrix metalloproteinase-9 (MMP-9) in the blood and the activities of glutathione peroxidase (GSH-Px), superoxide dismutase (SOD), and catalase (CAT).

A significant weak negative correlation was found between the MMP-9 serum level and SOD activity, with a correlation coefficient (r) of -0.419 (P-value=0.0071). In contrast, a significant strong negative correlation was observed between the MMP-9 serum level and GSH-Px activity, with an r value of -0.791 (P-value=0.0004), indicating a strong inverse relationship between the two variables. Moreover, a strong positive relationship was identified between the MMP-9 serum level and CAT activity, with an r value of 0.893 (P-value<0.0001). Additionally, a significant moderate positive correlation between the SOD activity and GSH-Px activity, with an r value of 0.529 (P-value <0.0001) was observed indicating a positive relationship between the two variables. Furthermore, a weak negative relationship was identified between the SOD activity and CAT activity, with an r value of -0.377 (P-value=0.0164), suggesting a weak inverse relationship. Finally, a significant moderate negative correlation was observed between the GSH-Px activity and CAT activity, with an r value of -0.686 (P-value<0.0001, Figure-3).

Histopathological Examination of the Intestine

H&E staining of intestine sections of control negative group I shows normal intestinal structure with villi and crypts lined by columnar epithelium with brush border and goblet cells. Group III is infected with E. coli 1×107 CFU, showing an abscess in the villus and intense inflammatory cell in the lamina propria, and decreased goblet cells. Group IV infected E. coli treated GA group showing resolve of the inflammation and restoration of normal intestinal architecture (Figure-4).

## Discussion

E. coli is a group of bacteria that can cause infections in the gut (GI tract), urinary tract and other body parts. Typically, it resides in the colon without causing any problems. Nevertheless, some strains can cause fever, vomiting, and watery diarrhea. The intestinal barrier is destroyed by the pathogenicity of E. coli, which also raises inflammatory factor levels and intestinal permeability. These actions cause diarrhea and the outflow of macromolecular substances [31]. The intestinal tract can become colonized by numerous pathogenic bacteria and inflamed once the intestinal microbial barrier is breached [32].

The animal gut is the body's largest immune organ and is vital for absorption and digestion [33]. It can strengthen immunity and shield the body from foreign pathogenic microbes as a vital interaction site between the body and the outside world [34]. Alterations in the intestinal barrier impact the absorption of nutrients and permit the entry of toxic substances [35].

This study’s findings revealed that group III mice infected with E. coli showed an increased rate of the serum Il-6 level above the normal range, almost double. E. coli infection, and this may be due to the activation of the immune response. IL-6 is a type of protein produced by immune cells in response to infection and tissue damage, playing a key role in the body's inflammatory response. E. coli triggers an immune reaction that stimulates the production and release of IL-6, which serves as a marker of the severity of the infection [36]. Several reports have confirmed this effect related to the elevation of such cytokine in different animals, as established by He et al. and Fayyaz et al. [37][38]. However, in group IV infected with E. coli and treated by GA, the serum Il-6 level was reduced and very close to the normal healthy range. This finding aligns with Khmaladze et al [39], who revealed the beneficial healthy effects of GA on inflammation by acting as an antioxidant, scavenging free radicals that contribute to oxidative stress and inflammation. This notable outcome, with a significant p-value of <0.0001, verifies that GA tremendously reduces inflammation and may be used as a therapeutic intervention for inflammatory conditions. This also accords with earlier observations, by Liu et al. [40] who claimed that GA helped the immune system for better response to inflammation.

Regarding Matrix Metalloproteinase (MMP)-9, a crucial regulator of inflammation and fibrosis in cardiovascular disease [41], group III E. coli infected mice exhibited a substantial increase in MMP-9 levels, exceeding normal range group I significantly P<0.0001. However, the beneficial effects of GA were apparent in group IV—mice infected with E. coli and treated with GA— where MMP-9 levels decreased noticeably to a range that closely resembled group I significantly P<0.0001. Therefore, GA may have preventive effects, reduce MMP-9 expressions and inhibit oxidative stress. This finding is in harmony with Bellioglu et al. [42]. These results may assume that the effects observed were not solely due to the antioxidant properties of GA but may also be attributed to GA’s role in hindering the growth of E. coli. This result aligns with the findings of Tian et al. [43]. Further, to investigate the positive impact of GA on E. coli, this study also examined GA's effect on several biomarker activities (i.e., SOD, GSH-Px, CAT), which play crucial roles in the body's antioxidant defense mechanism, as they help to preserve the level of reactive oxygen species (ROS) and shield cells from oxidative damage. Previous studies have shown that SOD has therapeutic potential against various diseases due to a deficiency in ROS levels, while CAT protects cells from oxidative damage [44][45]. E. coli and its lipopolysaccharide (LPS) can produce bad results that can interact with these enzymes’ healthy effects in the gut and manipulate their beneficial role [46]. This study, fortunately, revealed a strong correlation between the presence of GA and these biomarker functions. Mice in group III, infected with E. coli, showed a significant decrease in SOD and GSH-Px levels, as well as a threefold increase in CAT levels. However, this negative effect was significantly reversed in group IV, treated with GA, indicating that GA mitigated E. coli’s harmful effects, and this outcome is in agreement with Santos and Finlay [47].

This study’s results suggest that GA achieved its effects by enhancing the body's natural defense mechanisms in the mucous membranes, while also reducing the production of harmful factors. Additionally, GA may exert its influence by activating antioxidant pathways in the intestinal tissue and inhibiting the formation of toxic oxidants. Furthermore, GA's potent properties may be attributed to its capacity to neutralize ROS, including hypochlorous acid, hydrogen peroxide, hydroxyl radicals, and superoxide anions.

In fact, GA may help to reduce the inflammation in the colon by blocking certain proteins and preventing them from going into the cell's nucleus. This could be helpful for treating inflammatory conditions by stopping certain proteins from causing inflammation as was identified in an earlier study [48].

Furthermore, the immunohistochemical expression of IL-6 and TNF-α on the intestinal sections in the examined mice, groups I and II, showed very mild immunoreactivity in the basal layer of mucosa. However, group III exploring intense immunoreactivity in the whole mucosa were observed. The presence of E. coli in group III induced immunoreactivity in the epithelium and lamina propria, leading to inflammation and tissue damage, as shown in Figures-1 and -2. This pathogenicity depends on the adherence of the organism to the epithelial cells of the gastrointestinal tract and the initiation of the immune response. The response involves the release of pro-inflammatory cytokines, recruitment of immune cells, and activation of various immune pathways, causing a disruption in the epithelial barrier and infiltration of immune cells into the lamina propria, which can result in related symptoms such as diarrhea and abdominal pain [49].

These results are similar to Brauner et al.’s [50], who mentioned that the proximal tubular cultured cells were stimulated by E. coli exposure, resulting in significantly increased incidences of IL-6 and IL-8 expressing cells versus non-stimulated culture cells. Also, similar to Albrecht et al. [51], who claimed that the pathogen-induced by bacterial effects on host cells would activate pro-inflammatory cytokines like IL-6 and TNF-α. Contrastingly, our findings in group IV, where GA was administered as a treatment, differed from the other groups. There was a clear improvement in the intestine conditions, as revealed in our results.

The GA found in the natural plant material Radix Sanguisorbae has been shown to reduce the levels of IL-6, a key indicator of inflammation. Additionally, this compound has been found to decrease the activity of two key enzymes: Myeloperoxsidase and p-STAT3. These enzymes play a role in suppressing the expression of certain genes (i.e., TNF-α, IL-6, iNOS, and COX-2) [52]. The data results are similar to Pandurangan et al., who demonstrated that the protective mechanism of GA is also in the intestine and may be attributed to its capacity to prevent inflammation in dextran sulfate sodium (DSS)-induced colitis in mice. This healing effect may be achieved by inhibiting the activation of immune cells and subsequent inflammation in the intestinal tissues [53]. Additionally, GA supplementation increased the levels of microbiome or beneficial bacteria (i.e., Lactobacillus plantarum, Lactobacillus reuteri, Lactobacillus spp., and Lactobacillus lactis) resulting in an accompanying increase in fecal butyric acid production as affirmed by Kim et al. [54]. All the above factors could contribute individually or in combination to the improvement effect that helped restore the integrity of the epithelial barrier, prevent further disruption caused by E. coli itself or its harmful substances, and improve intestinal health. In the group of mice infected with E. coli, significant damage to the gut was observed, specifically the villi showing signs of abscesses, which are pockets of infected tissue. The surrounding area, the lamina propria, was filled with an intense accumulation of inflammatory cells. Additionally, the number of goblet cells, which are important for producing mucus, was significantly reduced.

In this area, a cluster of polymorphonuclear leucocytes was observed surrounded by a thin layer of mononuclear phagocytes. This cluster was surrounded by an intense accumulation of inflammatory cells (Figure-3C).

Lastly, the histopathological examination of the mouse intestine in different study groups revealed no histopathological changes in groups I and II, as shown in (Figure-3 A, B). Group III, infected with E. coli, showed abscess formation in the villus. The latter can be described as a central region of polymorphonuclear leucocytes, often with a thin mononuclear phagocyte infiltration surrounding it and intense inflammatory cell infiltration (Figure-3 C). These results are parallel to those of Savkovic et al., who revealed an increased number of lamina propria neutrophils with occasional intraepithelial lymphocytes, goblet cells, and crypt abscesses in the intestine of E. coli-infected animal samples [55].

This finding agrees with Kaper et al., who observed that E. coli infections are linked to a distinct intestinal histopathology known as 'attaching and effacing.' This phenomenon involves the intimate attachment of the bacteria to intestinal epithelial cells, resulting in significant cytoskeletal alterations. One notable change is the accumulation of polymerized actin directly beneath the adherent bacteria. Additionally, the microvilli of the intestine undergo effacement, while pedestal-like structures frequently emerge from the epithelial cells, providing a platform for the bacteria to perch on [56].

Controversy, group IV infected E. coli treated GA (Figure-3 D), showing no inflammation in intestine tissue with restoration of normal intestinal architecture. The findings of this investigation are in line with those reported by Lin et al [57], who clearly reported the potent ability of GA to work against bacteria other than E. coli, such as Klebsiella pneumoniae, another genus of the Enterobacteriaceae family, where GA was protective against the bactericidal activity of the Capsular polysaccharide (CPS) which can harm the immune system. Moreover, another interesting finding, similar to Lee et al.’s, who affirmed that GA also shows significant activity against bacteria other than Gram-negatives, displaying both antioxidant and antibacterial activity, suggesting its possibility to be used as an antibacterial agent for removing methicillin-resistant Staphylococcus aureus (MRSA) [58]. It must be stated, however, that discussing the differences between the mechanisms of GA in affecting these different bacteria is beyond the aim of this paper. Thus, to sum up, this study’s findings indicate that GA demonstrates potent bactericidal activity against E. coli, making it a promising natural compound for combating E. coli infections, inflammation, and tissue damage.

Study Limitations

Despite the insightful findings regarding anti-inflammatory and antioxidant effects of GA against E. coli infection, this study is not without limitations. One of the main ones is its relatively small subject pool. A larger sample size would offer stronger results and increase the generalizability of the findings.

However, animal models, although widely used in research, may not always accurately replicate human biological processes and responses to treatments. Another limitation is that the study utilized only male mice to exclude sex-related differences.

However, neglecting to include female subjects may have limited the generalizability of the findings. Furthermore, the study was conducted for a period of 21 days, which may not be sufficient to observe long-term effects of E. coli infection and GA treatment. A longer study duration could provide a more comprehensive understanding of the outcomes. Prolonged study periods may allow for a more comprehensive assessment of the effects being investigated. Moreover, another limitation, the study focused specifically on E. coli infection, using a single strain for experimentation. Including multiple bacterial strains or pathogens could provide a more nuanced and detailed understanding of the effects of GA on different bacterial infections.

In addition to the latter, the study primarily focused on biochemical, histopathological, and immunohistochemical parameters. However, including additional outcome measures, such as inflammatory cytokine profiles, microbiome analysis, or gene expression studies, could offer a more comprehensive understanding of the mechanisms involved. Finally, the study did not investigate dose-response relationships of GA in treating E. coli infections. Understanding the optimal dose and its effects could provide insights into the therapeutic potential of GA. Overall, while this research provides interesting insights into the impact of GA on E. coli infections, it is essential to consider its limitations when interpreting the results and planning future studies.

## Conclusion

This research aimed to assess the pathological and immunological consequences of E. coli infection and investigate the anti-inflammatory and antioxidant effects of GA against E. coli infection. It employed Swiss male mice as the experimental model and assigned them to four distinct groups for investigation purposes. Group III, which received only E. coli infection, showed lower antioxidant activity in the intestine and higher levels of serum biomarkers associated with inflammation than the control group.

Histopathological analysis revealed intestinal damage and inflammation in this group. However, group IV, receiving GA treatment alongside E. coli infection, exhibited improved biochemical, histopathological, and immunohistochemical outcomes, with reduced intestinal damage caused by E. coli. These findings suggest that GA has notable anti-inflammatory properties and can protect against intestinal injury caused by E. coli. Although previous research has investigated the therapeutic potential of GA, the current study innovatively examines the dose-dependent upregulation of a novel parameter in response to GA treatment, which has been previously unreported to the knowledge of the researcher. This parameter plays a crucial role in inflammation and is a critical factor in the development of effective treatment strategies. Moreover, this study uniquely explores the effects of GA treatment in an infected setting, providing valuable insights into its potential as a therapeutic agent against infection-induced intestinal damage. The decision to employ GA as a single agent, without any adjuvants, allows for a more accurate understanding of its effects and encourages further investigation into its mechanisms of action. Most notably, the study findings suggest that GA treatment may inhibit the apoptosis of intestinal cells, thereby offering a novel anti-apoptotic strategy against infection-induced intestinal damage. Such findings have significant implications for the development of effective treatments for some kinds of infections. Further research should focus on understanding the specific mechanisms underlying GA's protective effects against E. coli pathogenesis and its effect on human health.

## Acknowledgement

I would like to thank Dr. Osama A. Elkashty, assistant professor of Oral Pathology, Faculty of Dentistry, Mansoura University for his assistance in the histopathological part of this study.

## Conflicts of Interest

None.
